# Evaluation of injectable robenacoxib for the treatment of post-operative pain in cats: results of a randomized, masked, placebo-controlled clinical trial

**DOI:** 10.1186/s12917-016-0827-0

**Published:** 2016-09-29

**Authors:** Stephen King, Elizabeth S. Roberts, Jonathan N. King

**Affiliations:** 1 Elanco Animal Health, Companion Animal Development, Greensboro, NC 27408 USA; 2 Elanco Animal Health, Companion Animal Development, Basel, CH-4058 Switzerland

**Keywords:** Cat, Injectable, Post-operative, Pain, NSAID, Randomized, Robenacoxib

## Abstract

**Background:**

Few pharmaceuticals are registered in cats for the management of post-operative pain and inflammation. The objective of this study was to assess the field efficacy and safety of an injectable formulation of the nonsteroidal anti-inflammatory drug robenacoxib in cats undergoing surgery. The study was a multi-center, prospective, randomized, masked, parallel-group, placebo-controlled clinical trial. A total of 349 cats were enrolled and underwent surgery comprising forelimb onychectomy, as an example of orthopedic surgery, plus either ovariohysterectomy or castration. All cats received butorphanol prior to anesthesia and forelimb four-point regional nerve blocks with bupivacaine after induction of general anesthesia. Cats were randomized to receive daily subcutaneous (s.c.) injection of robenacoxib, at a target dosage of 2.0 mg/kg (*n* = 174), or placebo (*n* = 175) once prior to surgery and for an additional two days post-operatively.

**Results:**

Significantly (*P* = 0.037) fewer cats administered robenacoxib received additional analgesia rescue therapy (34 of 173, 19.7 %) compared to cats given placebo (73 of 175, 41.7 %). The percentage of treatment success was therefore 80.3 % with robenacoxib and 58.3 % with placebo. Behavior, posture, pain on palpation of the paw and soft tissue surgery sites, and overall pain were significantly (*P* < 0.05) improved versus placebo at various time points within the first 8 h in cats receiving robenacoxib. The most frequently reported adverse events were incision site infection/dehiscence, bleeding, vomiting, decreased appetite and lethargy. Frequencies of reported adverse clinical signs, hematology, serum chemistry and urinalysis variables, and body weight changes were similar between groups. There were no significant changes from baseline with robenacoxib in hepatic, hematological or renal clinical pathology variables.

**Conclusions:**

Robenacoxib by s.c. injection was effective and well tolerated in the control of post-operative pain associated with orthopedic, ovariohysterectomy and castration surgery in cats.

## Background

Pain management has been used increasingly as the standard of practice in cats undergoing surgery [1, 2]. In addition to improving animal welfare, control of post-operative pain and inflammation facilitates the healing process and helps avoid the development of chronic pain [3].

Opioids and nonsteroidal anti-inflammatory drugs (NSAID) are the classes of drugs used most frequently for controlling pain and inflammation in the immediate post-operative period. However, only few NSAIDs are licensed for use in cats, probably due to the relatively poor safety profile of several NSAIDs in this species [4].

Robenacoxib is a coxib class NSAID that demonstrates high selectivity for the cyclo-oxygenase-2 enzyme [5]. Robenacoxib injection (2 mg/kg body weight by the subcutaneous (s.c.) route) and tablets (1 mg/kg with a range 1-2.4 mg/kg) are registered in cats for up to three days use for the treatment of pain and inflammation associated with orthopedic or soft tissue surgery in the EU (www.ema.europa.eu) [6] and for the control of post-operative pain and inflammation associated with orthopedic surgery, ovariohysterectomy and castration in the US (www.fda.gov/Drugs). Randomized, masked, non-inferiority clinical studies demonstrated superior efficacy of injectable robenacoxib in reducing post-operative pain compared to meloxicam in a Japanese study [7] and non-inferior efficacy in an EU study [8]. Superior efficacy of oral robenacoxib in reducing post-operative pain compared to placebo was reported in a previous US field study [9].

The objective of the present US study was to demonstrate the field effectiveness and safety of injectable robenacoxib at a dose of 2.0 mg/kg for the control of post-operative pain associated with onychectomy, ovariohysterectomy and castration surgery in cats.

## Methods

### Study design

The study was a multi-center, randomized, masked, placebo-controlled, parallel-group clinical trial at 13 companion animal veterinary clinics located at various geographic locations within the US.

The study was conducted in accordance with guidelines for Good Clinical Practice (VICH GL9), Adequate and Well-controlled Studies (21 CFR 514.117) and New Animal Drugs for Investigational Use (21 CFR 511.1) (www.fda.gov/AnimalVeterinary/GuidanceComplianceEnforcement/). The protocol was reviewed and approved by the Food and Drug Administration (FDA) and Novartis Animal Health Institutional Animal Care and Use Committee. All owners provided written consent at the pre-enrollment visit (Day -14 to -2) for their cat to enter the study.

This manuscript was prepared in compliance with the CONSORT guidelines on randomized trials [10].

### Selection criteria

Clinically normal intact cats that were ≥4 months of age and weighing between 2.5 and 12 kg (inclusive) at the time of enrollment and presented to the clinic for ovariohysterectomy or castration plus forelimb onychectomy (declaw) were included.

Exclusion criteria were cats meeting any of the following criteria:

- pregnant;
- uncontrolled endocrine or systemic disorders (diabetes mellitus or hyperthyroidism had to be stabilized for at least 28 days prior to inclusion);
- history of concurrent diseases involving coagulation or circulatory or integumentary systems, distal limbs, gastrointestinal tract, kidney or liver;
- surgical intervention within 2 weeks of screening;
- requiring additional procedures that might interfere with assessments of pain;
- treated with alternative forms of pain relief (e.g., chiropractic manipulation, dry or wet acupuncture, acupressure, clinical therapy) within 30 days, a topical or systemic anti-inflammatory product such as an NSAID within 14 days, a short-acting (systemic or local) corticosteroid within 30 days, or long-acting corticosteroids within 60 days before inclusion into the study;
- known intolerance to any of the anesthetics used during the study; and
- known to be fractious, aggressive or frightened in a veterinary practice

### Anesthesia and analgesia protocol

With the exception of xylazine and medetomidine (which have analgesic properties), any combination of available products to facilitate induction, maintenance and recovery from anesthesia was allowed. All cats were adequately hydrated prior to and during surgery.

To provide a minimum level of pain control, all cats received butorphanol at a dose of 0.4 mg/kg body weight s.c. as an anesthetic pre-medication, followed by a metacarpal four-point ring block with bupivacaine (0.5 %) under aseptic conditions to provide local anesthesia [11]. The total dose of bupivacaine for both paws did not exceed 5.0 mg per kg body weight. The following nerves were blocked: median nerve, palmar branches of the ulnar nerve, dorsal digital nerves II to V and dorsal digital nerve I [9].

### Randomization and treatment

Cats selected for the study were formally included on Day 0 and randomly allocated to one of the treatment groups in a 1:1 ratio in blocks of four. Cats were administered either the injectable form of robenacoxib s.c. at a dosage of 2 mg/kg of body weight (Onsior®, Elanco Animal Health, Greenfield, US) or 0.1 mL/kg placebo (0.9 % sodium chloride injection, USP) once daily for 3 days. The dosage of 2 mg/kg was determined from a laboratory kaolin model study [12] and was confirmed in field studies in Japan and the EU [7, 8]. The dose administered to each cat was calculated from the pre-anesthetic body weight determined on Day 0. The first treatment was given approximately 30 min prior to surgery or at the same time the pre-anesthetic agents were administered. Subsequent once daily injections were given at approximately the same time each day.

The randomization list was computer-generated by the statistician. Masking was maintained as robenacoxib for injection was similar in appearance to the placebo. In addition, a treatment administrator (i.e. dispenser) at each clinic was responsible for dispensation and reconciliation of used and unused products. All study site personnel were masked to treatment assignment except the dispenser.

### Clinical examinations and follow-up

Clinical examinations were performed prior to enrollment and on Day 0 (prior to surgery) and Day 2 (study exit), in cases of early withdrawal, and for any animal which experienced a serious adverse event (SAE). The examination included a routine assessment of general appearance, body weight and major systems.

### Premature completion and follow-up

Cats could be withdrawn from the study and/or receive rescue analgesic therapy at any time at the discretion of the veterinarian. Cats receiving rescue intervention were observed in the clinic for a minimum of 24 h post-intervention and any potential adverse events (AEs) were documented. The owners of study cats received a follow-up phone call approximately 3 to 7 days after normal or premature completion to assess the animal’s general well-being.

### Rescue therapy and prohibited concomitant medications

If at any time the veterinarian determined a cat to be uncomfortable or in pain, the cat received additional butorphanol tartrate or any other product selected by the investigator (except other NSAIDs) to control pain as rescue analgesic therapy.

The following treatments were not allowed during the study: additional analgesic drugs or NSAIDs, synthetic feline facial pheromone (i.e., FELIWAY® Pheromone Spray), corticosteroids, α_2_-adrenoceptor agonists (e.g., medetomidine or xylazine) or alternative forms of pain relief (e.g., acupressure, dry or wet acupuncture, chiropractic manipulation, clinical therapy).

### Surgical procedures

Cats underwent onychectomy in addition to either castration or ovariohysterectomy. Onychectomy (declaw) was performed on the cats’ forelimbs (only) using one of the following procedures: guillotine nail trimmer, laser or surgical scalpel. Ovariohysterectomy was performed through a standard ventral midline incision. A flank approach, which was associated with higher wound pain in one study, was not allowed [13]. Castrations were performed through the standard scrotal approach. Cryptorchid cats were eligible and the surgical site was evaluated accordingly.

### Efficacy assessments

All efficacy assessments for each cat were made by the same veterinarian. The primary efficacy variable was the need for rescue therapy, decided by the veterinarian, to control post-operative pain (treatment failure) in the cats.

Secondary efficacy variables included the assessment of posture, behavior (viewed from a distance and determined following social interaction), pain elicited on palpation (paws and soft tissue incision site) and overall pain control (Appendix). A baseline evaluation of the secondary variables was performed on Day 0 after the cat had acclimatized for a minimum of 2 h in the clinic, and prior to administration of the test items or pre-anesthetic agents.

The evaluations for the primary and secondary variables were conducted at the time of post-surgical extubation (defined as 0 min) and thereafter at 30 min (±10 min); 1 h (±10 min); 3, 5 and 8 h (±15 min); 24, 28, 32, 48 and 52 h (±1 h).

### Safety assessments

Safety was assessed, in all cats which had received at least one dose of injectable robenacoxib or placebo, from all reported AEs, injection site reactions (warmth, visible swelling, palpation), post-study owner follow-up, changes in body weight and clinical pathology variables (hematology, serum chemistry and urinalysis).

### Statistical analysis

The study was planned to include a minimum of 300 cats, with 150 cats in each of the robenacoxib and placebo groups. All analyses were performed using SAS/STAT® software [14]. Unless stated otherwise, data are presented as mean (SD). Statistical significance was concluded with two-tailed *P* values less than 0.05. The experimental unit was each individual cat.

#### Primary efficacy variable

The primary efficacy variable was the frequency of rescue therapy (“rescue” or “treatment failure”), with superiority established by a statistically significant lower proportion of rescues in the robenacoxib group compared to the placebo group. A general linear model (PROC GLIMMIX) was used with fixed effect of ‘treatment’ and the random effects of ‘site’ and ‘treatment by site’. A logit link function was used because the analysis involved the binary variable ‘outcome’ (i.e., ‘success’ or ‘failure’). In addition, the ‘time to rescue’ for each cat was assessed from a Kaplan-Meier survival curve with comparison of groups using the log-rank, Cox-Tarone and Gehan-Breslow tests.

#### Secondary efficacy variables

The secondary efficacy variables (posture, behavior, pain elicited on palpation and overall pain control) were categorical and were measured multiple times during the study. Data on the day of surgery (extubation to 8 h) were compared statistically using PROC GLIMMIX. The model included the fixed effects of ‘treatment’, ‘time’ and ‘treatment by time’, the random effects of ‘site’, ‘treatment by site’ and ‘treatment by site and time’. The Last Observation Carried Forward (LOCF) method [15] was used up to 8 h post-extubation in order to take into account results from cats that received rescue therapy on the day of surgery. After 8 h, the LOCF method was not used, and results were not compared statistically, as the data became less meaningful due to (unequal) withdrawal of cases from the study.

Hematology, serum chemistry and urinalysis variables were evaluated statistically using analysis of covariance (ANCOVA; PROC MIXED) with the pre-treatment value as covariate. The model included the fixed effect of ‘treatment’ and random effects of ‘site’ and ‘treatment by site’. Levene’s test for homogeneity of variance was performed prior to ANCOVA. If the results from the Levene’s test were not significant (*P* > 0.01), untransformed data were used in the ANCOVA, and if significant (*P* ≤ 0.01) logarithmic, square root or reciprocal transformations were made.

The change in body weight from Day 0 to the end of the study (or exit) was analyzed using analysis of variance (ANOVA; PROC MIXED), with the fixed effects of ‘treatment’ and random effects of ‘site’ and ‘treatment by site’.

## Results

### Study cats

A total of 349 cats were enrolled in the study and all were included in the demographic and safety analysis, including reports of AEs: 174 cats received robenacoxib and 175 received placebo.

The cats were aged 4 months to 9 years and weighed 2.4 to 6.0 kg at enrollment (Day 0). Most cats (73.4 %) were aged ≤1 year and were in the weight range 2.5–3.4 kg (64.8 %). The most common breed was domestic short hair (74.8 % of cats) (Table 1). Similar numbers of males and females were enrolled within the groups; the percentages of cats undergoing ovariohysterectomy (52.3 and 54.9 %) and castration (47.7 and 45.1 %) were similar in the robenacoxib and the placebo groups, respectively.

**Table 1:** Demographic data of the cats

Variable	Robenacoxib (*n* = 174)		Placebo (*n* = 175)	
	*N*	%	*N*	%
Gender				
Female	91	52.3	96	54.9
Male	83	47.7	79	45.1
Breed				
Domestic short hair	133	76.4	128	73.1
Domestic medium hair	10	5.7	14	8.0
Domestic long hair	16	9.2	18	10.3
Himalayan	2	1.1	0	0.0
Manx	0	0.0	1	0.6
Persian	2	1.1	0	0.0
Ragdoll	3	1.7	2	1.1
Selkirk rex	0	0.0	1	0.6
Siamese	7	4.0	8	4.6
Siamese mix	1	0.6	3	1.7

Of the 349 cats enrolled in the study, 348 were analyzed for efficacy variables. One cat, which received robenacoxib, was not included because the animal died prior to surgery due to anesthetic equipment malfunction that was judged to be unrelated to the administration of robenacoxib.

### Primary efficacy variable

During the study, a total of 107 cats received rescue analgesic therapy with 34 of 173 cases (19.7 %) in the robenacoxib group compared to 73 of 175 cases (41.7 %) in the placebo group. The percentage of treatment success was therefore 80.3 % with robenacoxib and 58.3 % with placebo. Using Proc GLIMMIX, the difference between groups was statistically significant (*P* = 0.0370), with least squares mean estimates of 83.5 and 61.9 % treatment success for robenacoxib and placebo groups respectively.

The majority of rescues occurred at or before 8 h post-extubation, with 56/107 (52 %) at ≤ 3 h, 77/107 (72 %) at ≤ 5 h and 91/107 (85 %) at ≤ 8 h. The number of cats receiving rescue therapy at the 0, 0.5, 1, 3, 5, 8, 24 and 28 h time points (or in the interval since the previous time point) was respectively 0, 4, 6, 7, 4, 5, 7 and 1 in the robenacoxib group (total 34) and 0, 6, 11, 22, 17, 9, 6 and 2 in the placebo group (total 73).

In the robenacoxib group, the proportion of cats rescued was similar for guillotine-type nail trimmer (32.4 %), laser scalpel (32.4 %) and surgical (35.3 %) methods (Table 2). In the placebo group, however, more cats were rescued when onychectomy was performed by the guillotine-type nail trimmer (41.1 %) compared to the laser scalpel (28.8 %) and surgical (30.1 %) methods.

**Table 2:** Number of cats that received rescue analgesia according to surgical methods

Surgery/group	Surgery type	*N*	% of total
Onychectomy			
Robenacoxib	Guillotine-type nail trimmer	11	32.4 %
	Laser scalpel	11	32.4 %
	Surgical	12	35.3 %
	Total	34	
Placebo	Guillotine-type nail trimmer	30	41.1 %
	Laser scalpel	21	28.8 %
	Surgical	22	30.1 %
	Total	73	
Soft tissue surgery			
Robenacoxib	Castration	11	32.35 %
	Ovariohysterectomy	23	67.65 %
	Total	34	
Placebo	Castration	31	42.5 %
	Ovariohysterectomy^a^	42	57.5 %
	Total	73	

^a^Includes three female cats that were found to have already been neutered i.e. they underwent abdominal exploratory surgery

**Fig. 1:**
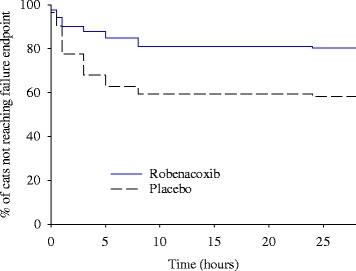
Kaplan-Meier plot of time to rescue analgesia therapy. The data show the percentage of cats at each time point which had not received rescue therapy to control post-operative pain (defined as treatment failure). Time 0 was fixed as the time of post-surgical extubation. There was a lower frequency of treatment failure in the robenacoxib group compared to the placebo at 30 min and all subsequent times. *P* < 0.0001 with log-rank, Cox-Tarone and Gehan-Breslow tests

In the survival analysis, the log-rank, Cox-Tarone and Gehan-Breslow tests were all highly significant (*P* < 0.0001) in favor of the robenacoxib group. The robenacoxib group had a lower probability of failures (rescue) at 30 min post-extubation and all subsequent remaining times compared to the placebo group (Fig. 1).

The most common reasons for administering rescue therapy were tenderness of surgical sites, agitation, aggressive or defensive/guarding behavior, hunched posture and vocalization (Table 3).

**Table 3:** Reasons for rescue analgesic therapy

Reason	Robenacoxib		Placebo	
	*N*	% of total (*n* = 34)	*N*	% of total (*n* = 73)
Tenderness of surgical sites	23	67.7 %	60	82.2 %
Agitated	21	61.8 %	46	63.0 %
Aggression or defensive/guarding behavior	21	61.8 %	38	52.1 %
Hunched posture	19	55.9 %	38	52.1 %
Vocalized response	18	52.9 %	36	49.3 %
Purposeful avoidance of painful stimulus	16	47.1 %	35	48.0 %
Chewing, licking, or biting of surgical sites	15	44.1 %	27	37.0 %
Little or no social response	12	35.3 %	13	17.8 %
Dilated pupils	10	29.4 %	14	19.2 %
Trembling or shaking	9	26.5 %	25	34.3 %
Tachycardia or tachypnea	8	23.5 %	10	13.7 %
Poor or unkempt appearance	5	14.7 %	1	1.4 %
Difficult or violent post anesthetic recovery	4	11.8 %	8	11.0 %
Other^a^	3	8.8 %	1	1.4 %

More than one reason may have been reported for each cat

^a^ “Other” included clinical signs that the investigator associated with pain, including licking lips, holding ears down/flat when palpated, breathing hard and not allowing an assessment of the paws

### Secondary efficacy variables

For the individual variables, the analysis showed statistically significant (*P* < 0.05) differences between groups and in favor of robenacoxib for behavior (from a distance and following social interaction), soft tissue incision site pain on palpation and overall pain score at assessment times 1, 3, 5 and 8 h; and posture and paw pain on palpation scores at 3, 5 and 8 h (Table 4).

**Table 4:** Summary statistics for secondary efficacy variables

Group	Time	*N*	Posture score		Behavior viewed from a distance score		Behavior following social interaction score		Paw pain on palpation score		Soft tissue incision site pain on palpation score		Overall pain control score	
			Mean (SD)	*P* value	Mean (SD)	*P* value	Mean (SD)	*P* value	Mean (SD)	*P* value	Mean (SD)	*P* value	Mean (SD)	*P* value
Robenacoxib	0	175	3.72 (0.83)	0.85	1.18 (0.51)	0.95	1.99 (1.19)	0.78	4.82 (0.49)	0.72	1.06 (0.27)	0.51	1.11 (0.35)	0.75
Placebo		173	3.70 (0.89)		1.17 (0.51)		1.97 (1.21)		4.86 (0.41)		1.02 (0.15)		1.09 (0.32)	
Robenacoxib	30 min	175	3.06 (1.06)	0.54	1.31 (0.55)	0.19	1.92 (1.04)	0.26	4.39 (0.96)	0.12	1.24 (0.55)	0.60	1.36 (0.56)	0.12
Placebo		173	2.99 (1.12)		1.23 (0.49)		1.80 (0.93)		4.56 (0.68)		1.21 (0.43)		1.25 (0.50	
Robenacoxib	1 h	175	2.65 (1.16)	0.24	1.35 (0.56)	**0.049**	1.88 (0.95)	**0.042**	4.19 (1.08)	0.052	1.37 (0.59)	**0.0067**	1.50 (0.65)	**0.0050**
Placebo		173	2.52 (1.18)		1.24 (0.49)		1.67 (0.89)		4.40 (0.93)		1.20 (0.47)		1.31 (0.59)	
Robenacoxib	3 h	175	2.32 (1.15)	**0.0022**	1.43 (0.61)	**0.0086**	1.81 (0.92)	**0.0079**	3.73 (1.27)	**<0.0001**	1.53 (0.73)	**0.0001**	1.66 (0.75)	**<0.0001**
Placebo		173	1.98 (1.07		1.27 (0.53)		1.53 (0.84)		4.22 (0.96)		1.30 (0.52)		1.37 (0.65)	
Robenacoxib	5 h	175	2.21 (1.07)	**0.0001**	1.49 (0.66)	**0.0001**	1.83 (0.94)	**0.0011**	3.57 (1.37)	**<0.0001**	1.60 (0.76)	**0.0076**	1.78 (0.82)	**<0.0001**
Placebo		173	1.78 (0.93)		1.26 (0.53)		1.50 (0.81)		4.02 (1.02)		1.44 (0.57)		1.43 (0.68)	
Robenacoxib	8 h	175	2.10 (1.07)	**0.0001**	1.50 (0.68)	**<0.0001**	1.81 (0.93)	**0.0006**	3.52 (1.33)	**0.0008**	1.65 (0.75)	**0.0017**	1.83 (0.82)	**<0.0001**
Placebo		173	1.66 (0.87)		1.20 (0.51)		1.45 (0.77)		3.88 (1.06)		1.46 (0.60)		1.43 (0.67)	
Robenacoxib	24 h	107	1.32 (0.61)	ND	1.07 (0.25)	ND	1.21 (0.49)	ND	4.11 (1.0)	ND	1.34 (0.58)	ND	1.18 (0.45)	ND
Placebo		146	1.36 (0.60)		1.05 (0.23)		1.25 (0.59)		3.99 (1.05)		1.39 (0.59)		1.22 (0.51)	
Robenacoxib	28 h	104	1.36 (0.67)	ND	1.04 (0.19)	ND	1.13 (0.39)	ND	4.16 (0.92)	ND	1.33 (0.57)	ND	1.16 (0.37)	ND
Placebo		140	1.26 0.54		1.04 (0.19)		1.19 (0.46)		3.99 (0.92)		1.37 (0.55)		1.16 (0.39)	
Robenacoxib	32 h	102	1.30 (0.63)	ND	1.02 (0.14)	ND	1.13 (0.36)	ND	4.14 (1.02)	ND	1.30 (0.56)	ND	1.13 (0.34)	ND
Placebo		139	1.24 (0.49)		1.02 (0.15)		1.17 (0.45)		4.01 (0.93)		1.37 (0.56)		1.17 (0.37)	
Robenacoxib	48 h	102	1.23 (0.51)	ND	1.01 (0.10)	ND	1.10 (0.33)	ND	4.06 (0.98)	ND	1.26 (0.56)	ND	1.12 (0.32)	ND
Placebo		139	1.15 (0.38)		1.01 (0.12)		1.09 (0.36)		3.96 (0.95)		1.28 (0.50)		1.07 (0.26)	
Robenacoxib	52 h	102	1.20 (0.47)	ND	1.01 (0.10)	ND	1.11 (0.34)	ND	4.11 (0.92)	ND	1.28 (0.55)	ND	1.10 (0.30)	ND
Placebo		139	1.12 (0.35)		1.02 (0.15)		1.09 (0.36)		4.07 (0.94)		1.27 (0.52)		1.06 (0.24)	

*P* values less than 0.05 are shown in bold. Statistical analyses were performed only on data from time 0 min through hour 8

Last Observation Carried Forward (LOCF) was applied for secondary variables from 0 to 8 h for cats which received rescue therapy (but not for 24 to 52 h)

*ND* not done

### Safety – adverse events

Adverse events and post-study phone findings were reported in 26 of 174 cases (14.9 %) in the robenacoxib group and 9 of 175 cases (5.1 %) in the placebo group. Table 5 provides a summary of the number and percentage of cases with each reported AE.

**Table 5:** Adverse events reported during and at post-study follow up

Adverse event	Robenacoxib (*n* = 174)^a^		Placebo (*n* = 175)	
	*N*	% of total	*N*	% of total
Incision site infection, dehiscence	9	5.2	0	0
Increased incision site bleeding	6	3.4	4	2.3
Vomiting	5	2.9	0	0
Decreased appetite	4	2.3	3	1.7
Lethargy (after day of surgery)	4	2.3	2	1.1
Urinary tract infection	2	1.1	0	0
Coughing	1	0.6	0	0
Fever	1	0.6	0	0
Semiconscious^b^	1	0.6	0	0
Soft stool or diarrhea	0	0	2	1.1

Cats may have experienced more than one type or occurrences of an event

^a^Not including one cat treated with robenacoxib which suffered cardiopulmonary failure after a pneumothorax was caused by failure of the non-rebreathing equipment shortly after intubation

^b^Semiconscious cat full recovered

The most common AEs reported in cats treated with robenacoxib were incision site infection/dehiscence, increased incision site bleeding, vomiting, decreased appetite and lethargy. In the placebo group the most common AEs reported were increased incision site bleeding and decreased appetite.

During the study, one cat in the robenacoxib treatment group died due to cardiopulmonary failure prior to surgery as a result of an anesthesia equipment malfunction that caused pneumothorax. The cat was excluded from the efficacy and clinical pathological analyses. One death (placebo group) was reported following the completion of the study; the cat died from an obstructed urethra resulting in kidney failure and presumed heart failure due to elevated potassium concentrations.

### Safety - clinical pathology

Selected kidney, liver and hematology variables at the study exit are shown in Table 6.

**Table 6:** Selected kidney, liver and hematological variables at study exit

Variable (Laboratory reference range)	Robenacoxib (*n* = 170)			Placebo (*n* = 172)			*P* value
	Mean (SD)	*N* ^a^		Mean (SD)	*N* ^a^		
		Higher	Lower		Higher	Lower	
Serum							
Urea nitrogen, mg/dL (14–36 mg/dL)	23.5 (6.58)	6	2	22.1 (5.82)	3	2	0.21
Creatinine, mg/dL (0.6–2.4 mg/dL)	0.88 (0.19)	0	2	0.94 (0.32)	1	6	0.093
Alkaline phosphatase, U/L (6–102 U/L)	48.5 (25.8)	5	0	52.5 (31.3)	14	0	0.10
Alanine aminotransferase, U/L (10–100 U/L)	52.1 (19.1)	5	4	58.6 (44.7)	8	3	0.26
Aspartate aminotransferase, U/L (10–100 U/L)	32.7 (21.7)	3	0	31.3 (18.1)	3	0	0.59
Total bilirubin, mg/dL (0.1–0.4 mg/dL)	0.11 (0.031)	0	0	0.11 (0.043)	1	0	0.54
Total protein, g/dL (5.2–8.8 g/dL)	6.91 (0.63)	1	0	6.95 (0.55)	0	0	0.34
Albumin, g/dL (2.5–3.9 g/dL)	3.44 (0.36)	11	1	3.44 (0.34)	8	1	0.47
Hematology							
Hemoglobin, g/dL (9.3–15.9 g/dL)	12.1 (1.57)	1	5	11.9 (1.74)	0	10	0.49
Hematocrit, % (29–48 %)	37.0 (5.19)	6	8	36.4 (5.57)	1	18	0.39
Platelet count, 10^3^/μL (200–500 10^3^/μL)	275.4 (105.0)	5	39	290.8 (109.5)	6	31	0.51
Urine^b^							
Urine specific gravity (1.015–1.060)	1.067 (0.018)	109	0	1.058 (0.019)	79	6	**0.014**

*P* value less than 0.05 is shown in bold

^a^Number of cats with value for respective variable higher or lower than the reference range at study exit

^b^For urine, *n* = 160 for both robenacoxib and placebo

Mean values for all serum chemistry variables in both treatment groups were within normal reference ranges with the exception that creatine phosphokinase at study exit in both the robenacoxib (1468.0 U/L) and placebo (1151.8 U/L) groups was higher than the upper limit (529 U/L) of the normal range.

The blood urea nitrogen to creatinine ratio, chloride and triglyceride values were significantly higher at study exit in the robenacoxib compared to the placebo group (*P* = 0.016, 0.032, and 0.018, respectively). The glucose values were significantly lower in the robenacoxib compared to the placebo group (*P* = 0.0086).

Mean values for all hematology variables at pre-treatment and study exit were within the normal reference range in both groups with the exception of the absolute neutrophil counts at study exit (8589.4 cells/μL) for the placebo group, which were slightly higher than the upper limit (8500 cells/μL) of the normal range. The percentage of lymphocytes was significantly higher in the robenacoxib compared to the placebo group (*P* = 0.031); however, the difference in the absolute lymphocyte counts was not statistically significant (*P* = 0.078).

### Safety - body weight

There was no significant (*P* = 0.11) difference in change from baseline in body weight between the robenacoxib and placebo groups (−0.027 [0.13] and −0.05 [0.13] kg, respectively).

### Safety - injection site reaction

One cat in the robenacoxib group had a mild swelling at the Day 2 injection site, which was observed at study exit. Another cat in the robenacoxib group had “greasy hair” at the Day 2 injection site that was observed at study exit. A further cat in the placebo group had “blood around injection site” for the Day 0 injection site that was observed on Day 2.

## Discussion

In this clinical trial of cats undergoing forelimb onychectomy in combination with ovariohysterectomy or castration, all animals received pre-surgical analgesia with butorphanol and a regional nerve block. Addition of robenacoxib by s.c. injection, approximately 30 min prior to surgery and then once daily for two subsequent days, was well tolerated and provided better control of post-operative pain compared to placebo. The superior efficacy of robenacoxib compared to placebo was evidenced from the significantly (*P* = 0.037) lower frequency of rescue therapy (19.7 % versus 41.7 %, the primary endpoint), which is consistent with a treatment success rate of 80.3 % versus 58.3 %. In addition, robenacoxib demonstrated superior efficacy to the placebo for the secondary endpoints of posture, behavior assessed from a distance and during social interaction, as well as pain assessed at the paw, soft tissue incision site and overall. Similar results were obtained previously with robenacoxib tablets [9], and in both studies once daily dosing with robenacoxib by injection or tablets provided effective analgesia over the 24 h dosing interval. These results support previous findings that robenacoxib has a longer duration of action than would be predicted from its short blood half-life, explained by concentration and persistence at sites of inflammation [16, 17]. The endpoints included in this study did not include specific measures of inflammation. Inhibition of inflammation, pain and fever by robenacoxib was demonstrated previously in an experimental kaolin model in cats [12].

Onychectomy is a model of orthopedic surgery and has been used in several studies for testing analgesics and NSAIDs in cats, either alone or in combination with neutering [11, 18–21]. In this study, therefore, cats underwent both orthopedic (onychectomy) and soft tissue (ovariohysterectomy or castration) surgeries. The frequency of rescue analgesia was 19.7 % with robenacoxib and 41.7 % with placebo, used in addition to pre-surgery butorphanol and bupivacaine nerve blocks. A previous study using an identical methodology reported similar results with robenacoxib tablets (16.5 %) compared to placebo (46.3 %) [9]. Other historical studies in cats undergoing onychectomy with or without neutering reported the following frequencies of rescue therapy: 95 % (negative control) and 17 % (pre and post-surgery butorphanol) [11]; 67 and 71 % with single pre-surgery meloxicam and butorphanol, respectively [20]; and 27 % (transdermal fentanyl patch) and 9 % (butorphanol) [18]. In none of these studies did cats receive pre-surgery local anesthesia, although one study did not show any additional analgesic benefit of a four-point regional nerve block with bupivacaine, when added to buprenorphine [21]. In a fourth study, no rescue therapy was administered to cats undergoing only onychectomy after receiving butorphanol or transdermal fentanyl [19].

This study was a comparison of injectable robenacoxib to a placebo. Administration of placebo to animals in pain studies raises ethical and welfare issues; however, these were overcome by providing both butorphanol and regional bupivacaine nerve blocks to all cats prior to surgery. The efficacy of butorphanol has been demonstrated compared to a negative control in cats undergoing onychectomy, with or without neutering, at an intramuscular dosage of 0.2 mg/kg, lower than the 0.4 mg/kg dosage used in this study [11]. Local nerve block with bupivacaine has also been tested in cats undergoing onychectomy; however, no significant benefit when added to buprenorphine was reported in one study [21]. A 185 min duration of action was reported for intramuscular administration of 0.4 mg/kg butorphanol using the thermal threshold method in healthy cats [22]. The results from this study suggest that butorphanol and bupivacaine did not have strong efficacy, however, since rescue therapy was administered in most cases at early time points. Of the 107 cats which received rescue analgesia, 56 (52)%, 77 (72 %) and 91 (85 %) were rescued on or before 3, 5 or 8 h, respectively. In order to minimize suffering, any cat could be withdrawn from the study and administered rescue analgesia at any time at the discretion of the veterinarian.

The most frequently reported AEs were incision site infection/dehiscence, increased incision site bleeding, vomiting, decreased appetite and lethargy. Other studies have reported that 50 % of onychectomy surgeries have complications including pain, bleeding and lameness, regardless of onychectomy technique [23, 24]. The frequency of incision site infection/dehiscence and vomiting was higher in the robenacoxib than the placebo group. Similar findings were reported previously with robenacoxib tablets in cats undergoing the same surgeries [9]. It is not known if these results are reliable, however, as vomiting was not observed in safety studies with robenacoxib, even with repeated administration of high doses [16], and the impact of NSAIDs on wound healing in humans was concluded not be clinically relevant [25].

There was no evidence from this study of any toxicity of robenacoxib to target organs that are most sensitive to NSAID toxicity (gastrointestinal tract, kidney and liver), consistent with previous studies in cats [16, 26]. At daily dosages up to 20 mg/kg for 42 days, oral robenacoxib was well tolerated and had no detectable effect on clinical chemistry, coagulation or hematology variables in healthy young cats [16]. In a field study in cats with osteoarthritis, robenacoxib tablets at a dose of 1–2.4 mg/kg once daily for one month were well tolerated with no evidence of gastrointestinal, kidney or liver toxicity [26].

The main limitations of this study are discussed below. First, no specific criteria were pre-defined for the use of rescue analgesia therapy; the veterinarians could rescue cats at any time they thought additional analgesics were necessary. The stated reasons for administering rescue therapy were in all cases consistent with pain (Table 3), however, and were most frequently reported as tenderness of surgical sites, agitation, aggressive or defensive/guarding behavior, hunched posture and vocalization.

Second, analysis of the secondary efficacy endpoints was challenging due to the unequal frequency of withdrawal of cases after administration of rescue therapy between the two groups with the placebo group having more rescues than those cats treated with robenacoxib. The secondary efficacy data for the first 8 h were therefore analyzed using the LOCF method. The LOCF method [15] has limitations, but was justified in this study since it was used only for cases proactively withdrawn due to lack of efficacy and for a limited period (up to 8 h post-extubation).

## Conclusions

Robenacoxib by s.c. injection at a target dose of 2.0 mg/kg once daily for three days was effective and well tolerated in the control of post-operative pain in cats undergoing orthopedic plus ovariohysterectomy or castration surgeries.

## Appendix

Summary of secondary efficacy variables

**Table:**

Secondary efficacy variable	Description	Scale
Posture	Cat’s overall mobility within the cage (standing or resting position), any preferential or unequal weight distribution of the limbs, hunched or retracted posture, position of head and any forelimb shifting behavior.	•Normal •Mildly abnormal •Moderately abnormal •Severely abnormal
Behavior	Cat’s overall comfort, response to social interaction with examiner and / or hospital staff, level of aggression, level of vocalization, and ease of handling as viewed from a distance and following social interaction.	Behavior as viewed from a distance •Appears comfortable •Questionable comfort •Distressed animal Behavior following social interaction •Normal •Mildly abnormal •Moderately abnormal •Severely abnormal
Pain on palpation	Cat’s level of response to a gradual increase in pressure applied to areas adjacent to the surgical sites. The response was the amount of pressure that elicited any level of pain response from the cat (e.g., withdrawal of paw, discomfort or vocalization).	Paw onychectomy site (pressure was assessed using Palpometer®). Response was based on the audio feedback: •5 beeps (greatest recorded pressure) •4 beeps •3 beeps •2 beeps •1 beep (lightest recorded pressure) Castration or ovariohysterectomy wound Response was based on subjective evaluation •Significant pressure •Moderate pressure •Slight pressure
Overall pain control	Subjective assessment of overall pain control.	•Well controlled •Moderately controlled •Poorly controlled

Palpometer® is a device strapped to the index finger which measures pressure applied to a given area (Palpometer®, University of Victoria Innovation and Development Corp., PO Box 3075 STN CSC, R Hut McKenzie Avenue, Victoria, BC, Canada, V8W 3 W2). Scores of 1, 2, 3, 4 and 5 beeps correspond respectively to pressures of 200, 300, 450, 600 and 800 gf/cm^2^

All devices were calibrated before use

## Abbreviations

AE: Adverse event
ANCOVA: Analysis of covariance
ANOVA: Analysis of variance
EU: European Union
FDA: Food and Drug Administration
GLIMMIX: General Linear Model Mixed
LOCF: Last Observation Carried Forward
NSAID: Nonsteroidal anti-inflammatory drug
s.c.: Subcutaneous
SAE: Serious adverse event
US: United States
